# High expression of *SPP1* in patients with chronic obstructive pulmonary disease (COPD) is correlated with increased risk of lung cancer

**DOI:** 10.1002/2211-5463.13127

**Published:** 2021-03-07

**Authors:** Ti‐wei Miao, Wei Xiao, Long‐yi Du, Bing Mao, Wei Huang, Xue‐mei Chen, Cong Li, Yan Wang, Juan‐juan Fu

**Affiliations:** ^1^ Respiratory Group Department of Integrated Traditional Chinese and Western Medicine West China Hospital Sichuan University Chengdu China; ^2^ West China Biobanks Department of Clinical Research Management West China Hospital Sichuan University Chengdu China; ^3^ Research Core Facility West China Hospital Sichuan University Chengdu China

**Keywords:** bioinformatics, chronic obstructive pulmonary disease, lung cancer, meta‐analysis, *SPP1*

## Abstract

Chronic obstructive pulmonary disease (COPD) is characterized by persistent airway inflammation and fixed airflow obstruction. Patients with COPD have increased risk of lung cancer (LC), and the coexistence of both diseases is associated with poorer survival. However, the mechanisms predisposing patients with COPD to LC development and poor prognosis remain unclear. Gene expression profiles were downloaded from the Gene Expression Omnibus. Twenty‐two data sets were included (*n* = 876). We identified 133 DEGs and 145 DEGs in patients with COPD and LC compared with healthy controls, respectively. There were 1544 DEGs in patients with LC and coexisting COPD compared with COPD, and these DEGs are mainly involved in the cell cycle, DNA replication, p53 signalling and insulin signalling. The biological processes primarily associated with these DEGs are oxidation reduction and apoptosis. *SPP1* was the only overlapping DEG that was up‐regulated in patients with COPD and/or LC, and this was validated by qPCR in an independent cohort. The area under the curve value for *SPP1* was 0.893 (0.822–0.963) for the prediction of LC in patients with COPD. High expression of *SPP1* in patients with LC was associated with shorter survival time. Up‐regulation of *SPP1* may be associated with increased risk of LC in patients with COPD and therefore may have potential as a therapeutic target for LC in patients with COPD.

## Graphical abstract

*SPP1* is progressively up‐regulated in the lungs of patients with COPD, lung cancer (LC) and LC coexisting with COPD, as shown by bioinformatic analysis. Up‐regulation of *SPP1* expression in COPD and its further up‐regulation in NSCLC coexisting with COPD were confirmed by qPCR. The area under the curve value for *SPP1* was 0.893 (0.822–0.963) for the prediction of LC in patients with COPD. Thus, up‐regulation of *SPP1* may be associated with increased risk of LC in patients with COPD.

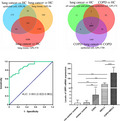

**Abbreviations**
AUC: area under the curve
CI: confidence intervals
COPD: chronic obstructive pulmonary disease
DAVID: Database for Annotation, Visualization and Integrated Discovery
DEGs: differentially expressed genes
FDR: false discovery rate
GEO: Gene Expression Omnibus
GO: gene ontology
GSEA: Gene Set Enrichment Analysis
IGF: insulin growth factor
NSCLC: non‐small‐cell lung cancer
OPN: osteopontin
qPCR: quantitative real‐time polymerase chain reaction
ROC: receiver operating characteristic curve
*SPP1*: secreted phosphoprotein 1

Chronic obstructive pulmonary disease (COPD) and lung cancer (LC) are leading cause of morbidity and mortality worldwide. COPD is characterized by persistent airway inflammation and fixed airflow obstruction [1]. The overall prevalence of spirometry‐defined COPD is 8.6% among the general population aged 20 years or older, accounting for 99.9 million people with COPD in China [2]. It is currently the third leading cause of death worldwide and imposes huge economic burden on patients [3]. LC is the number one cause of cancer deaths worldwide with a low 5‐year overall survival rate [4, 5]. A previous study has reported that the prevalence of COPD was 40–70% of those diagnosed with LC [6, 7]. The incidence of these two diseases has been increasing in recent years, and this trend is estimated to continue. More importantly, both conditions often coexist such that patients with COPD have an increased risk of developing LC resulting in a poorer prognosis compared with LC alone [8].

The mechanisms underlying the coexistence of COPD and LC are not well understood. One of the main hypotheses is the common environmental risk factor for both diseases in particular cigarette smoke exposure. However, smokers with COPD are fourfold to sixfold as likely to develop LC as smokers without COPD [9], and the presence of emphysema in COPD predicts a higher LC risk adjusted for smoking status [10, 11]. Therefore, the presence of COPD itself is an independent risk factor for LC. In terms of prognoses, a series of studies have demonstrated that patients with non‐small‐cell lung cancer (NSCLC) coexisting with COPD were related to worse survival and complained of more symptoms [12, 13, 14, 15]. Potential mechanisms linking COPD and LC may be associated with shared pathogenic processes such as increased oxidative stress and DNA damage, repression of the DNA repair mechanisms, chronic exposure to pro‐inflammatory cytokines, repression of innate immunity and increased cellular proliferation [16]. Nevertheless, the molecular basis that predisposes patients with COPD to LC and is related to worse survival remains unclear. Therefore, it is important to identify potential pathological genes and pathways involved.

With the development of microarray technology, gene expression analysis using high‐throughput platforms has been widely applied to explore the differentially expressed genes (DEGs) within various diseases. A group of gene expression profiling studies on COPD and LC have been performed using microarray technology and identified certain DEGs associated with COPD and LC [17, 18, 19]. However, the DEGs identified with microarray in a single study depend on sample size, technology platform and sample types, etc., while the overlapping genes obtained from multiple microarray data sets might be more accurate and representative. There is no study addressing DEGs and pathways related to the coexistence of COPD and LC using meta‐analysis of combined data sets so far.

In the present study, we interrogated the Gene Expression Omnibus (GEO) database for multiple microarray expression data sets and compared mRNA expression among different disease groups in terms of COPD, LC and LC coexisting with COPD, aiming to identify molecular signatures, significant biological functions and pathways associated with the coexistence of COPD and LC. More importantly, results were further validated by quantitative real‐time polymerase chain reaction (qPCR) and analysed for the association with survival.

## Material and methods

### Data collection

All data sets in the current study were searched in the GEO database (http://www.ncbi.nlm.nih.gov/geo/) by using key words as follows: [(COPD or chronic obstructive pulmonary disease or emphysema or chronic bronchitis or LC or lung squamous cell carcinoma or lung adenocarcinoma or NSCLC or lung or epithelial) AND (smok^*^ or cigarette) AND human]. Original studies were further searched in PubMed to ensure the inclusion of all relevant data sets by using key words: [(COPD or chronic obstructive pulmonary disease or emphysema or chronic bronchitis or LC or lung squamous cell carcinoma or lung adenocarcinoma or NSCLC or lung or epithelial) AND (mRNA or messenger RNA or gene) AND (smok^*^ or cigarette) AND human].

The inclusion criteria for data sets were as follows: (a) having screened for DEGs related to COPD and LC by using human biological samples; (b) available information of smoking history for each sample; (c) available results of pulmonary function test if there was no description of COPD diagnosis in study design; (d) available raw data or matrix file in GEO database; and (e) data sets in which samples were not completely overlapped with other data sets.

The inclusion criteria for patients were as follows: (a) available information for smoking history; (b) with data of lung function tests or information for the diagnosis of COPD; and (c) available raw data or matrix file of mRNA expression on airway epithelial cell sample or lung tissue sample. COPD was defined in patients with pulmonary function test of FEV_1_ (forced exhalation volume in one second) /FVC (forced vital capacity) < 0.7 or with doctor‐diagnosed COPD in the data set. LC was confirmed by pathological diagnosis. The exclusion criteria were as follows: (a) unavailable information for smoking history or non‐smoker; (b) overlapping samples in other data sets that have been included in current study; (c) other sample types such as blood; and (d) with respiratory diseases other than COPD and LC.

### Identification of DEGs

Data sets were merged and analysed only when the experimental platforms and sample types were identical. Limma package in r software (version 3.5.1, https://www.r‐project.org/) was applied to perform the normalization of raw data or matrix file, and log_2_ conversion of fold change for each disease group. Genes with absolute fold change ≥ 1.5 in expression level and adjusted *P* value < 0.05 were considered as statistically significant DEGs when two groups were compared. Ggplot2 package in r software was used to perform the volcano plots of specific genes. Gplots package in r software was applied to perform the heatmap of top 50 DEGs (top 25 up‐regulated genes and top 25 down‐regulated genes). Venn diagram package in r software was applied to identify the overlapping DEGs.

### Functional annotation analysis

Database for Annotation, Visualization and Integrated Discovery (DAVID, https://david.ncifcrf.gov), an online tool, was used to analyse gene ontology (GO) of DEGs. In detail, GO depicts three complementary biological concepts including biological process, molecular function and cellular component. *P* < 0.05 was regarded as an accepted threshold criterion.

### Gene Set Enrichment Analysis (GSEA)

Gene Set Enrichment Analysis is a computational method that determines whether an a priori defined set of genes shows statistically significant and concordant differences between two biological states [20]. GSEA was applied to perform Kyoto Encyclopedia of Genes and Genomes pathway analysis. Data of gene expression, phenotype, gene sets that compose GSEA were input to GSEA software (version 3.0) to run pathway analysis. The number of permutations was 1000. Gene sets in GSEA with false discovery rate (FDR) < 0.25 and nominal *P* < 0.05 were considered statistically significant.

### The associations between key gene and clinical outcomes

Receiver operating characteristic curve (ROC) analysis was performed to investigate the role of key gene in the prediction of coexisting LC in COPD, and the area under the curve (AUC) value with 95% confidence intervals (CI) was calculated. The levels of key gene were compared by different GOLD grades and by different stages of LC in patients with COPD and in those with LC, respectively.

The Kaplan–Meier plotter database is capable of assessing the effect of 54 000 genes on survival in 21 cancer types, which includes survival analysis data of 3452 patients with LC (http://kmplot.com/analysis/index.php?p=background). Survival analysis of the selected key gene was performed by the Kaplan–Meier plotter database. Hazard ratios (HR) with 95% CI and log‑rank *P* values were calculated and displayed.

### RNA extraction and qPCR validation

Lung tissue specimens were obtained from patients who underwent lobectomy or pneumonectomy for cancer in West China Hospital. After surgical resection, lung tissues were immediately frozen to −80 °C. Histologically normal tissues adjacent to the tumour were used as controls. This study was approved by the Clinical Trial and Biomedical Ethics Committee of West China Hospital of Sichuan University (No.2016‐120) and was conducted in accordance with the principles of Declaration of Helsinki. All patients volunteered to attend the study and signed an informed consent. Total RNA was extracted from lung tissues (nine non‐smoker controls, 15 smoker controls, 13 COPD, eight NSCLC and 16 NSCLC coexisting with COPD) according to the manufacturer's protocol using the E.Z.N.A. HP Total RNA Kit (OMEGA, GA, USA). iScript cDNA Synthesis Kit (Bio‐Rad, Hercules, CA, USA) was applied to synthesize cDNA following the manufacturer's instructions. qPCR was performed in triplicate by using iQ™ SYBR Green Supermix (Bio‐Rad) according to the manufacturer's protocol. Relative expression level of gene was normalized via the GAPDH *C*_t_ value (endogenous reference), applying a $2^{‐\Delta \Delta C_{t}}$ relative quantification method. The qPCR primers were as follows:

*SPP1*‐forward: 5′‐CAACAAATACCCAGATGCTGTGGC‐3′.

*SPP1*‐reverse: 5′‐GGACTTACTTGGAAGGGTCTGTGG‐3′.

*GAPDH*‐forward: 5′‐TGCACCACCAACTGCTTAGC‐3′.

*GAPDH*‐reverse: 5′‐GGCATGGACTGTGGTCATGAG‐3′.

### Statistical analysis

Statistical analysis was performed with spss version 22 (IBM Corporation, Chicago, IL, USA). Levels of mRNA expression were expressed as median (interquartile range) or mean ± standard deviation according to data distributed type. Comparisons between two different groups were determined by Student's *t*‐test for parametric data or Mann–Whitney test for nonparametric data. One‐way analysis of variance was used for multiple comparisons. *P* < 0.05 was considered statistically significant.

## Results

### Data collection

There were 79 data sets that were found with two different types of tissue (airway epithelial cell and lung tissue) based on 18 platforms. According to the inclusion criteria, 22 data sets (*n* = 876) based on three platforms (GPL570, GPL96 and GPL1708) were finally included in the current study for analysis (Table 1). To control the impact of smoking status, only smokers in the disease groups were included for analysis. The median age and number of male|female of the patients are listed in Table S1.

**Table 1:** Data sets included in the current study.

Tissue type	Platform	Dataset	Sample size			
			HC	LC	COPD	LC + COPD
Lung tissue	GPL570 [HG‐U133_Plus_2] Affymetrix Human Genome U133 Plus 2.0 Array	GSE43580, GSE31210, GSE37768	17	226	0	0
	GPL96 [HG‐U133A] Affymetrix Human Genome U133A Array	GSE10072	151	42	0	0
Airway epithelial cell	GPL570 [HG‐U133_Plus_2] Affymetrix Human Genome U133 Plus 2.0 Array	GSE64614, GSE43939, GSE5058 GSE13933, GSE19667, GSE63127 GSE8545, GSE22047, GSE20257 GSE11952, GSE30063, GSE11784 GSE11906, GSE10006	366	0	91	0
	GPL96 [HG‐U133A] Affymetrix Human Genome U133A Array	GSE994, GSE19027	26	21	0	0
	GPL1708 [Agilent‐012391 Whole Human Genome Oligo Microarray G4112A]	GSE12428, GSE12472	0	0	36	36

### Identification of DEGs

**Fig. 1:**
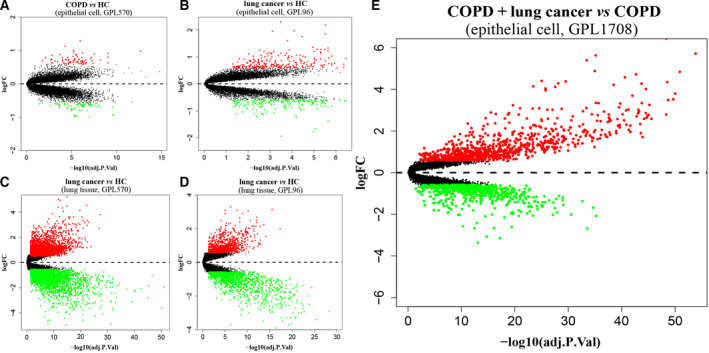
Volcano plots of DEGs. (A) COPD vs HC in epithelial cell of GPL570; (B) LC vs HC in epithelial cell of GPL96; (C) LC vs HC in lung tissue of GPL570; (D) LC vs HC in lung tissue of GPL96; (E) LC coexisting with COPD vs COPD in epithelial cell of GPL1708. Red and green dots denote up‐regulated and down‐regulated genes, respectively.

There were 133 DEGs (72 up‐regulated and 61 down‐regulated genes) when comparing COPD patients with healthy controls (HC; Fig. 1A). A total of 429 DEGs (232 up‐regulated and 197 down‐regulated genes) in LC patients were identified compared with HCs using samples of airway epithelial cells (Fig. 1B).

**Fig. 2:**
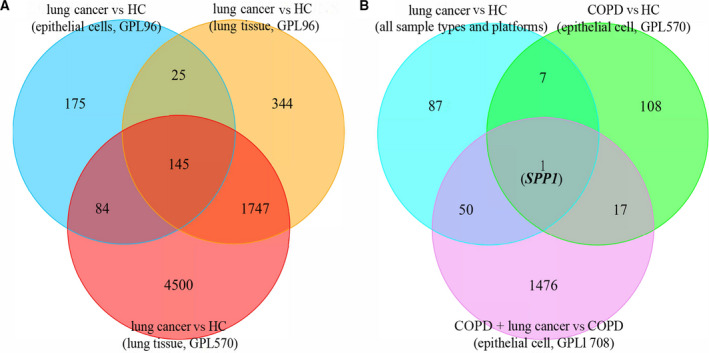
Venn diagrams of the DEGs. (A) The DEGs in LC vs HC across all sample types and platforms; (B) the overlapping DEG that was up‐regulated in patients with COPD, LC and LC coexisting with COPD.

**Fig. 3:**
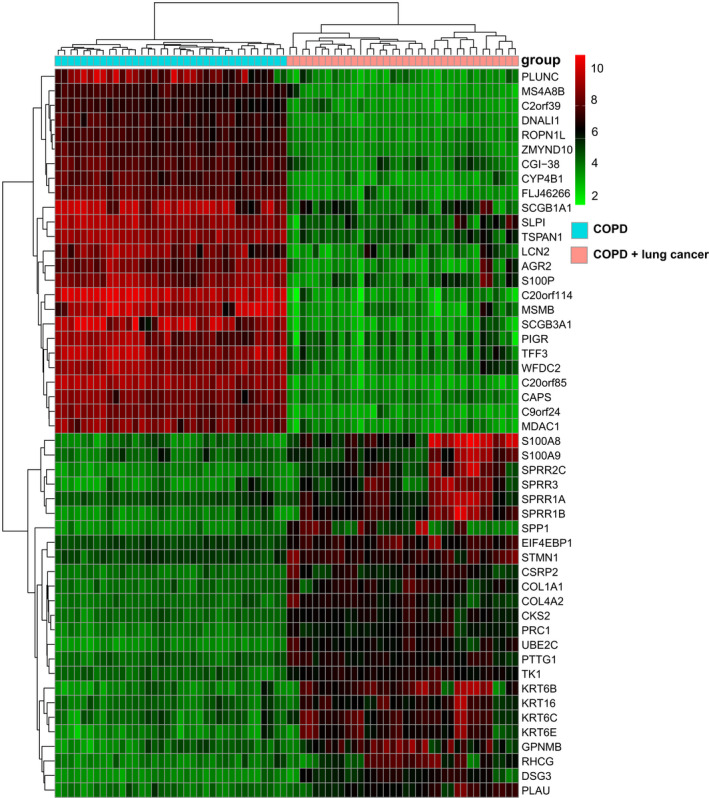
Heatmap of the top 50 DEGs (top 25 up‐regulated and 25 down‐regulated genes) in patients with LC and coexisting COPD vs COPD. Red indicates up‐regulation, and blue indicates down‐regulation.

By using lung tissue samples, there were 6476 DEGs (3307 up‐regulated and 3169 down‐regulated genes) in GPL570 and 2261 DEGs (954 up‐regulated and 1307 down‐regulated genes) in GPL96 when comparing LC patients with HCs (Fig. 1C,D). A total of 145 DEGs were confirmed in patients with LC across all sample types and platforms (Fig. 2A). There were 1544 DEGs (889 up‐regulated and 655 down‐regulated genes) in LC patients coexisting with COPD compared with COPD patients (Fig. 1E), and a heatmap was clustered by top 50 DEGs as shown in Fig. 3. The secreted phosphoprotein 1 (*SPP1*) was the only overlapping DEG that was up‐regulated in patients with COPD, LC and LC coexisting with COPD (Fig. 2B).

### Functional annotation analysis

**Fig. 4:**
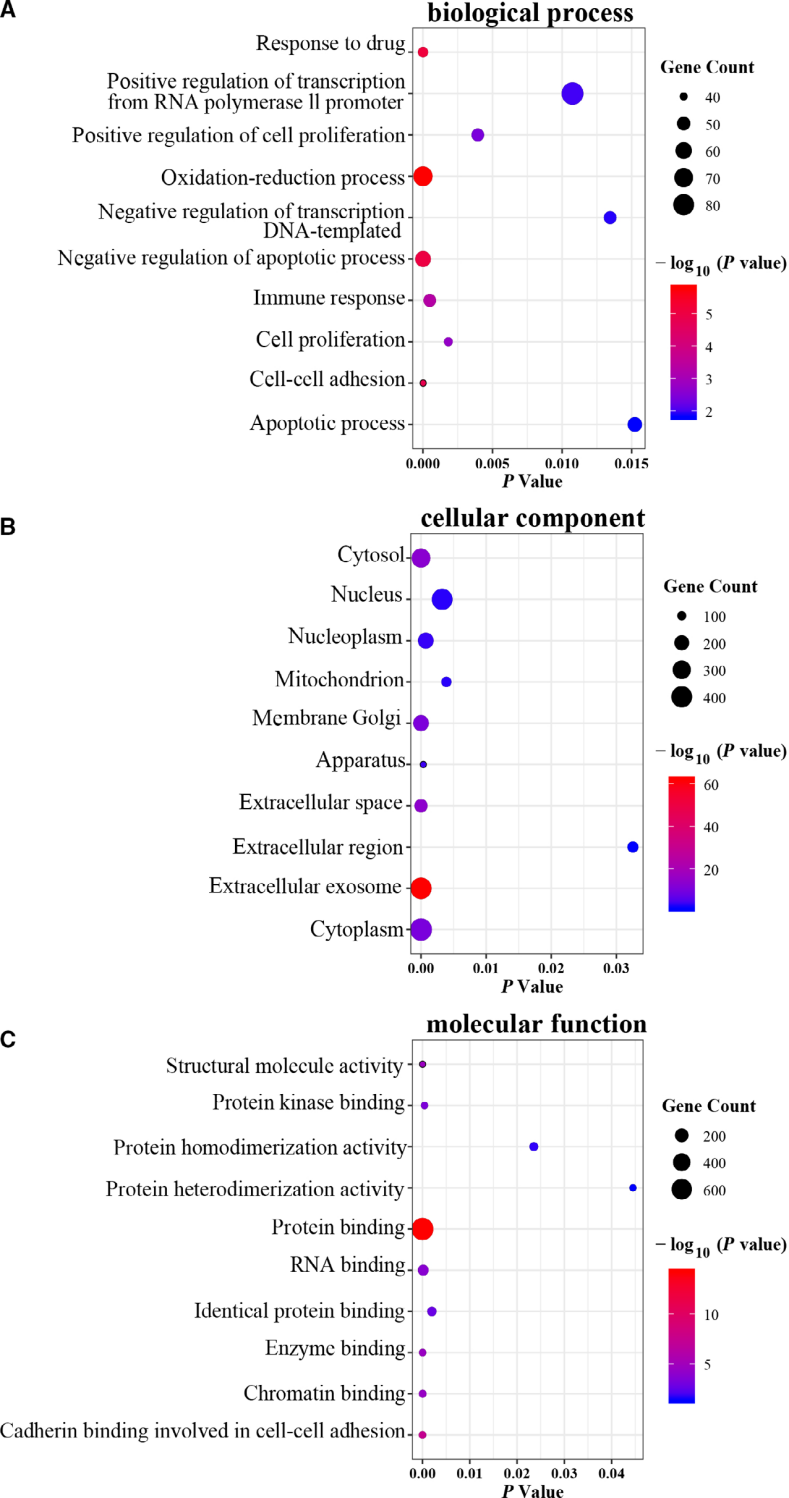
The top 10 significant enriched GO terms based on DEGs related to LC coexisting with COPD vs COPD by DAVID. (A) Biological process; (B) cellular component; (C) molecular function.

Functional enrichment analysis was performed with 1544 DEGs identified in the comparison of LC coexisting with COPD and COPD alone. The biological process was primarily associated with oxidation reduction process, positive regulation of transcription from RNA polymerase II promoter and apoptotic process (Fig. 4A). The cellular component mainly located in extracellular exosome, cytoplasm and cytosol (Fig. 4B). The molecular function was mainly related to protein binding, poly(A) RNA binding and identical protein binding (Fig. 4C).

### Gene Set Enrichment Analysis

The GSEA results showed that gene sets related to the groups of LC coexisting with COPD were mainly enriched in the pathways of cell cycle, P53 signalling pathway, DNA replication, pyrimidine metabolism, insulin signalling pathway, etc. (FDR < 0.25 and nominal *P* < 0.05; Table 2).

**Table 2:** GSEA of genes related to LC coexisting with COPD vs COPD (top 10 ranked according to *P* value).

Pathway name	NOM *P* value	FDR *q* value
Pyrimidine metabolism	< 0.001	0.035
Cell cycle	< 0.001	0.026
DNA replication	0.002	0.054
Pathways in cancer	0.01	0.025
Insulin signalling pathway	0.01	0.04
Fructose and mannose metabolism	0.017	0.037
p53 signalling pathway	0.017	0.048
RNA degradation	0.017	0.045
FC gamma r‐mediated phagocytosis	0.018	0.051
ECM–receptor interaction	0.022	0.046

### The associations between *SPP1* and clinical outcomes

**Fig. 5:**
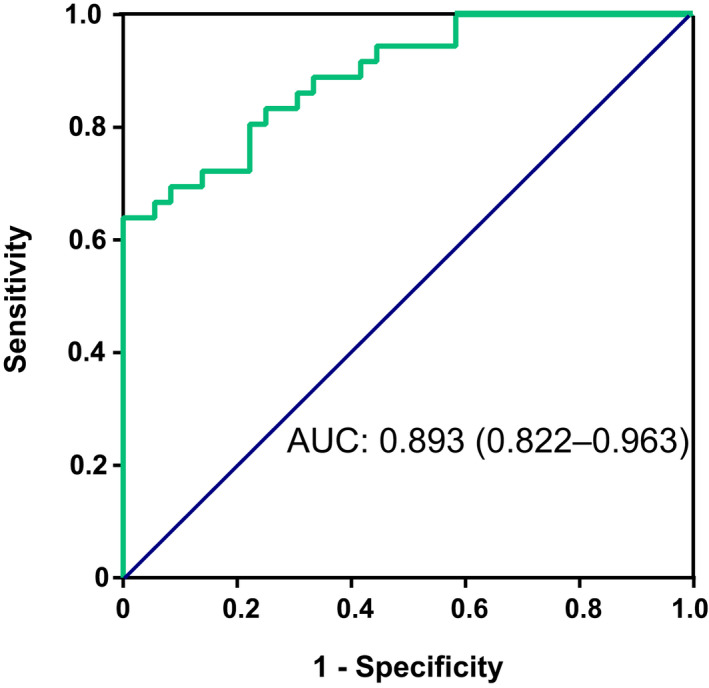
ROC analysis revealed that the relative expression levels of *SPP1* in the lungs could predict coexisting LC in patients with COPD, and AUC value with 95% CI was 0.893 (95% CI: 0.822–0.963).

The diagnostic value of relative expression levels of *SPP1* in the lungs for the prediction of coexisting LC in patients with COPD was evaluated by a ROC analysis. The AUC value with 95% CI for the relative expression levels of *SPP1* in the lungs was 0.893 (0.822–0.963), and the cut‐off value for the relative expression levels of *SPP1* in the lungs was 3.625 according to Youden's index with sensitivity of 80.56% and specificity of 77.78% (Fig. 5).

There were 91 patients with COPD across seven data sets in the bioinformatic analysis, in which 52 patients were reported with COPD severity. These patients were from the same platform (GPL570) with sample type of airway epithelial cells. There were 34 patients classified as GOLD stage 1, fifteen patients as GOLD stage 2 and three patients as GOLD stage 3, and the levels of *SPP1* expression were not significantly different among the three groups (*P* = 0.811).

The presence of advanced LC (stages IIIB and IV) is not suitable for surgery, we found few patients with stage III and IV LC in the bioinformatic analysis. Data of 226 lung tissue samples reporting the stage of LC were retrieved from platform GPL570, in which 129 samples were from patients with stage I LC and 97 samples were from patients with stage II LC. The levels of *SPP1* expression were statistically different between patients with stage I (11.74 ± 1.63) and stage II (12.16 ± 1.16; *P* = 0.030). Data of 42 lung tissues samples reporting the stage of LC were retrieved from platform GPL96, in which only two lung tissue samples were obtained from patients with stage IV. There was no significant difference in the levels of *SPP1* expression among patients with stage I (*n* = 16), stage II (*n* = 17) and stage III (*n* = 7) LC (*P* = 0.690).

**Fig. 6:**
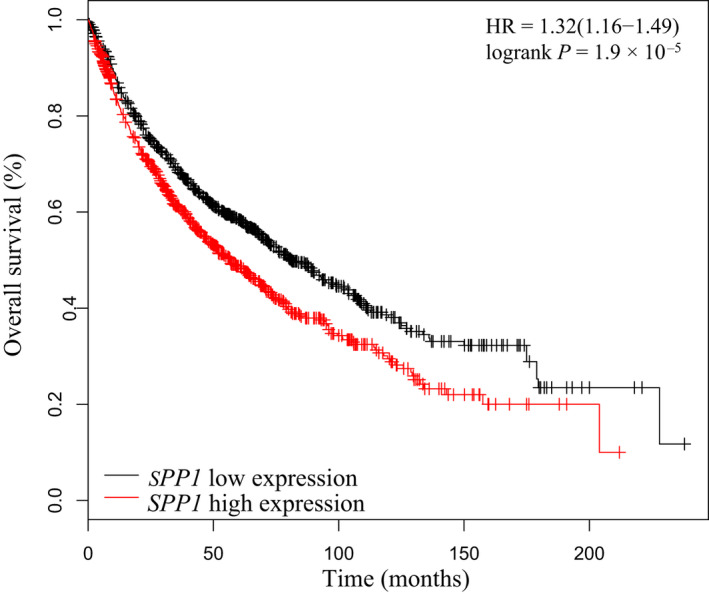
Survival analysis of *SPP1* in LC by using the Kaplan–Meier plotter database.

Survival analysis of the key gene *SPP1* was performed in 1926 patients diagnosed with LC using the Kaplan–Meier plotter database. Elevated level of *SPP1* expression was significantly associated with poor overall survival [HR = 1.32 (1.16–1.49); log‐rank *P* = 1.9 × 10^−5^] (Fig. 6).

### Results of qPCR validation

**Fig. 7:**
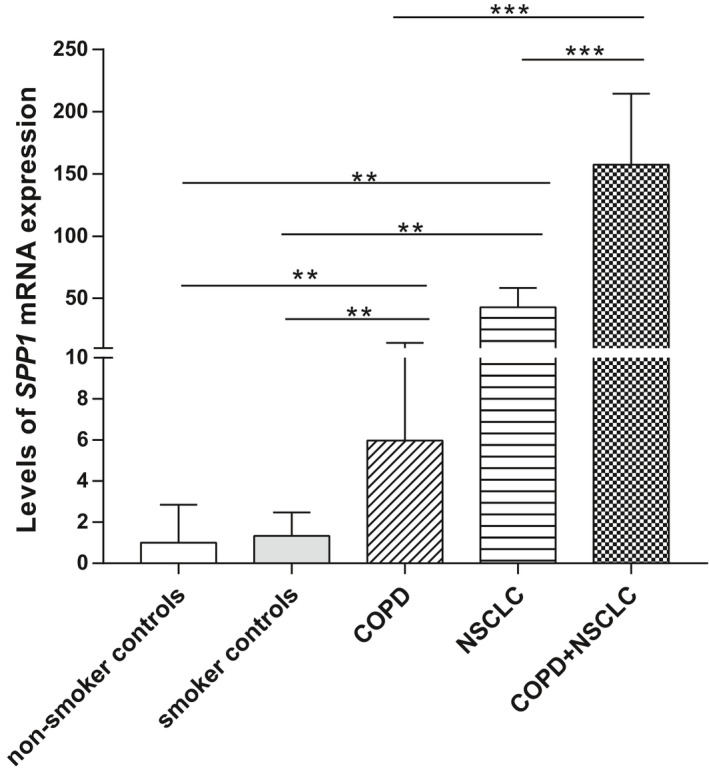
qPCR validation of SPP1 expression in a cancer cohort. Levels of SPP1 expression in lung tissues between non‐smoker controls (*n* = 9), smoker controls (*n* = 15), COPD (*n* = 13), NSCLC (*n* = 8) and NSCLC coexisting with COPD (*n* = 16) were compared by the Mann–Whitney test. Data are presented as median (interquartile range). **P < 0.01. ***P < 0.001.

To validate the results of bioinformatic analysis, the relative expression levels of gene *SPP1* were assayed in lung tissues using qPCR. The results showed that levels of *SPP1* expression in cancer tissues were higher than that in non‐smoker controls (*P* = 0.007) and smoker controls (*P* = 0.002). The expression level of *SPP1* in the COPD group was significantly higher compared with either non‐smokers (*P* = 0.002) or smokers (*P* = 0.001), and the level was further up‐regulated with an enormous increase in the group of NSCLC coexisting with COPD (*P* < 0.001). In addition, levels of *SPP1* expression were also significantly higher in the group of NSCLC and coexisting COPD than that in NSCLC group (*P* < 0.001; Fig. 7).

## Discussion

In this study, we firstly identified DEGs that were associated with COPD, LC and LC coexisting with COPD by bioinformatic analysis using multiple microarray expression data sets, in order to explore potential molecular mechanisms underlying the increased risk of LC in patients with COPD. Functional annotation analysis and GSEA were performed to investigate the potential roles of significant genes in the biological function and relevant pathways.

The most important finding of our study is the identification of the key gene *SPP1* in the coexistence of COPD and LC. *SPP1*, also named osteopontin (OPN), is a phosphorylated acidic glycoprotein that functions as both an extracellular matrix molecule and a cytokine [21]. The overexpression of the *SPP1* is associated with the growth and stage of LC, tumour angiogenesis and lymph node metastasis [22, 23, 24, 25, 26], and represents a more aggressive NSCLC type [23]. *SPP1* could also mediate macrophage polarization and immune escape in lung adenocarcinoma [27]. Besides, the tumour‐derived *SPP1* isoforms cooperate with both TRP53 and CCL2 and the FN1/*SPP1*‐ITGAV signalling chemoattracts tumour cells and inhibits their apoptosis, promoting the intrapulmonary metastasis of LC [28, 29]. Our study results of qPCR validation confirmed the up‐regulation of *SPP1* in patients with NSCLC, indicating its potential role as oncogene.

On the other hand, *SPP1* is associated with neutrophilic inflammation and emphysema [30]. Cigarette smoking can increase *SPP1* expression in induced sputum, and its level is increased in induced sputum [31] and lung tissue [32] of patients with COPD. Hence, *SPP1* may participate in the pathogenesis of COPD. By using bioinformatic analysis, we firstly found that *SPP1* was the only gene that differentially expressed in the comparisons of COPD vs HCs, LC vs HCs, and LC coexisting with COPD vs COPD alone in the lungs. Although the comparisons were based on different platforms and sample types, it may suggest a progressive up‐regulation of *SPP1* in COPD, LC and LC coexisting with COPD. We therefore conducted the qPCR validation, and the results confirmed that *SPP1* gene expression was significantly up‐regulated in COPD and further up‐regulated to a considerably higher level in the group of NSCLC coexisting with COPD. This suggests that as an oncogene, *SPP1* may participate in the predisposition of patients with COPD to LC development.

The above finding drives us to further investigate the clinical role of *SPP1* in predicting coexisting LC in COPD. The high AUC value of 0.893 for *SPP1* expression level indicated promising utility of *SPP1* as a biomarker in the lung for the prediction of LC risk in patients with COPD, although the sample size was not large enough. A previous study using serum OPN to predict cancer risk in patients with COPD identified a rather small AUC value of 0.636 [33]. There are two forms of OPN, intracellular form (iOPN) and a secreted form (sOPN) [34], and sOPN may be produced by many cell types, including hepatocytes, cholangiocytes, hepatic stellate cells, macrophages, T lymphocytes and natural killer T cells [35]. A previous study demonstrated that the lungs produce plasma OPN [36]; however, plasma OPN may partly originate from the lungs in COPD and LC because many other cells are capable of producing OPN. Hence, detecting *SPP1* gene or protein expression levels in local specimens such as lung tissue, BAL and sputum to predict coexisting LC in COPD is superior to plasma OPN and should be the direction of future research.

Previous studies have investigated the associations between *SPP1* and clinical outcomes in both COPD and LC. Patients with frequent exacerbations of COPD had higher plasma OPN than the non‐frequent exacerbators [37]. We did not find the association between *SPP1* and COPD severity, which was consistent with an early study [31]. Conflicting results exist in terms of cancer stage and *SPP1* level based on blood assay [38, 39]. We did not find the difference in the lungs using our data, which may be attributed to the somehow limited number of patients especially the limited number of patients with advanced LC, and this needs to be confirmed in following study. However, in terms of role of *SPP1* in clinical prognosis, the crucial role of *SPP1* found by our study was manifested by its association with low overall survival in LC. High *SPP1* expression in the tumour tissue was associated with inferior survival in a previous study with small sample size [24]. We confirmed its association with survival in LC by analysing data of 1926 patients in Kaplan–Meier plotter database. This indicates that the up‐regulation of *SPP1* expression may also be involved in the poor prognosis in terms of survival in patients with LC and coexisting COPD. Therefore, *SPP1* might be a therapeutic target to reduce the development of LC in patients with COPD and to improve survival time.

Several significant pathways were identified that related to the coexistence of LC with COPD. Cell cycle deregulation is a common feature of human cancer, and cancer cells frequently display unscheduled proliferation [40]. Cell cycle‐related and expression‐elevated protein in tumour overexpression is associated with cell proliferation and poor prognosis in NSCLC [41]. DNA replication occurs during the S (synthetic) phase of cell cycle [42] and may cause mutation and DNA damage [42]. Gene mutation has identified in squamous cell carcinoma and adenocarcinoma of lung [43, 44] and has led to resistance to chemotherapy, resulting in lower survival rates in small‐cell LC [45]. DNA damage is often associated with oxidative stress, resulting in cancer initiation [46]. Previous study has found that the insulin growth factor (IGF) pathway is activated in NSCLC [47]. Reduced levels of insulin‐like growth factor binding protein 3 in serum were associated with increased LC risk in current smokers independent of obesity [48]. Small molecule inhibitors targeting both the IR and IGF1R or blockading beta‐adrenergic receptor‐mediated insulin‐like growth factor receptor activation could reduce NSCLC cell proliferation and prevent LC [49, 50]. The p53 gene is the most frequently mutated gene in cancer [51], playing an important role in the regulation of tumour progression [52]. Meanwhile, p53 protein expression is significantly higher in patients with emphysema compared with healthy smokers or non‐smokers [53]. Our results further showed that the p53 signalling pathway was an important pathway related to the coexistence of COPD and LC. Thus, the p53 signalling pathway may participate in LC development in patients with COPD. Understanding of the pathways beyond significant molecules will provide mechanisms and research bases for the novel therapy, which needs to be addressed in future study.

Airway epithelial cells are extensively exposed to oxidants, causing both oxidative stress and inflammation, which contribute to COPD pathophysiology [54] and carcinogenesis [46]. We found that oxidation reduction was an important biological process related to patients with LC and coexisting COPD, which therefore may be involved in the increased risk of developing LC in patients with COPD. Apoptotic process was another important biological process linking the coexistence of COPD and LC in the current study. *SPP1* has been shown to participate in regulating apoptotic process in LC [28, 29]. Therefore, the apoptosis pathway might be the mechanism or pathway by which *SPP1* functions resulting in the development of LC in COPD and deserving further study.

There are some highlights in our study. It is the first study that investigated the molecular signatures and pathways underlying the coexistence of COPD and LC by integrating multiple mRNA microarray datasets and confirmed the DEGs in multi‐platforms using different tissue types from the lung, which provides more accurate and reliable results than a single study of microarray. Additionally, the results of bioinformatic analysis were validated by qPCR using lung tissues. Moreover, we identified the level of *SPP1* in the lungs for the prediction of coexisting LC in COPD for the first time. Finally, we confirmed the prognostic role of *SPP1* in the overall survival of patients with LC using data of 1926 patients from the Kaplan–Meier plotter database. The study also has certain limitations. We only used lung tissues from patients with NSCLC to reduce the effects of histological subtypes of LC on study results in validation study. However, squamous cell carcinoma was not classified, which is known to be more closely related to COPD, due to the limitation of sample size. The study results will be confirmed in future with larger sample size, and molecules and pathways involved in the upstream and downstream mechanism of *SPP1* deserve further study.

## Conclusion

In conclusion, our study identified the progressive up‐regulation of gene *SPP1* in the lungs of patients with COPD, LC and LC coexisting with COPD by using meta‐analysis of multiple microarray data sets with bioinformatic approach. Validation study confirmed the up‐regulation of *SPP1* expression in COPD and its further up‐regulation in NSCLC coexisting with COPD. Survival analysis showed that high expression of *SPP1* was associated with shorter survival time. The current study might indicate the roles of *SPP1* in the predisposition of patients with COPD to LC development. Lung *SPP1* might be a biomarker that could predict the coexistence of LC in COPD and the poorer prognosis of patients with coexisting COPD and LC. *SPP1* might be a therapeutic target to reduce the development of LC in patients with COPD and to improve survival time, which deserves further investigation.

## Conflict of interest

The authors declare no conflict of interest.

## Author contributions

T‐WM and WX collected and analysed data and drafted the manuscript. L‐YD, X‐MC, CL, WH and YW collected the samples, performed qPCR and drafted the manuscript. BM designed the experiment. J‐JF designed the experiment and revised the manuscript.

## Supporting information


**Table S1**. Demographic of the patients included for analysis.

## Data Availability

The data used to support the findings of this study are available from the corresponding author upon request.
