# Supplementary material for: High expression of SPP1 in patients with chronic obstructive pulmonary disease (COPD) is correlated with increased risk of lung cancer
Source: FEBS Open Bio. 2021 Mar 7;11(4):1237–49. doi: 10.1002/2211-5463.13127 (PMC8016137; doi:10.1002/2211-5463.13127)
Supplement: Supplementary file 1 — Table S1. Demographic of the patients included for analysis. [file FEB4-11-1237-s001.docx]

Table S1. Demographics of the patients included for analysis

| **Tissue type** | **Lung tissue** | | | | **Airway epithelial cell** | | | | | |
| --- | --- | --- | --- | --- | --- | --- | --- | --- | --- | --- |
| **Platform** | GPL570 | | GPL96 | | GPL570 | | GPL96 | | GPL1708 | |
| **Sample size** | HC | LC | HC | LC | HC | COPD | HC | LC | COPD | LC +COPD |
|  | 17 | 226 | 15 | 42 | 366 | 91 | 26 | 21 | 36 | 36 |
| **Age and sex**  **available** | 8(47.1%) | 226(100%) | 15(100%) | 42(100%) | 132(36.1%) | 54(59.3%) | 9(34.6%) | 21(100%) | 36(100%) | 36(100%) |
| **Age** | 58 (54.5-62.1) | 60.0 (53.8-65.0) | 68.0 (62.0-71.0) | 66.0 (59.0-73.0) | 39 .0 (35.0-47.0) | 51.5 (45.8-57.5) | 28.0 (25.0-49.0) | 66.0 (59.0-75.0) | 57.0 (54.0-66.0) | 66.5 (54.0-74.0) |
| **Sex** (**M/F**) | 4/4 | 194/32 | 4/11 | 31/11 | 87/45 | 41/13 | 6/3 | 17/4 | 24/12 | 34/2 |

HC=healthy control, LC=lung cancer, COPD=chronic obstructive pulmonary disease
